# Detecting the Causal Effect of Soil Moisture on Precipitation Using Convergent Cross Mapping

**DOI:** 10.1038/s41598-018-30669-2

**Published:** 2018-08-15

**Authors:** Yunqian Wang, Jing Yang, Yaning Chen, Philippe De Maeyer, Zhi Li, Weili Duan

**Affiliations:** 1 State Key Laboratory of Desert and Oasis Ecology, Xinjiang Institute of Ecology and Geography, Chinese Academy of Sciences, Urumqi, China; 2 University of Chinese Academy of Sciences, Beijing, China; 3 Department of Geography, Ghent University, Ghent, Belgium; 4 National Institute of Water and Atmospheric Research, Christchurch, New Zealand; 5 Sino-Belgian Joint Laboratory of Geo-Information, Urumqi, China; 6 Sino-Belgian Joint Laboratory of Geo-Information, Ghent, Belgium

## Abstract

As a vital land surface parameter, soil moisture influences climate through its impact on water and energy cycles. However, the effect of soil moisture on precipitation has been strongly debated. In this study, a new causal detection method, convergent cross mapping (CCM), was applied to explore the causality between soil moisture and precipitation over low- and mid- latitude regions in the Northern Hemisphere. CCM method generally identified a strong effect of soil moisture on precipitation. Specifically, the optimal effect of soil moisture on precipitation occurred with a lag of one month and clearly decreased after four months, suggesting that soil moisture has potentials to improve the accuracy of precipitation forecast at a sub-seasonal scale. In addition, as climate (i.e., aridity index) changed from dry to wet, the effect of soil moisture on precipitation first increased and then decreased with peaks in semi-arid and semi-humid areas. These findings statistically support the hypothesis that soil moisture impacts precipitation and also provide a reference for the design of climate prediction systems.

## Introduction

A growing body of evidence shows that variations in land surface conditions, particularly water storage^1^, vegetation^2^, and ice/snow cover^3^, can impact climate through the process of heat and water transfer between atmosphere and land surface. Soil moisture, a slowly varying surface parameter, can affect weather through its influence on energy and water fluxes^4,5^. Soil moisture-precipitation interaction is an important topic in the land-atmosphere coupling research, as it has the potential to improve the accuracy of precipitation prediction and the monitoring of extreme events such as floods and droughts^6,7^. Theoretically, the impact of soil moisture on precipitation can be dissected into two distinct parts: the impact of soil moisture on subsequent evapotranspiration, and the impact of evapotranspiration on subsequent precipitation^8^. The dependency of evapotranspiration on soil moisture is intuitive and easy to quantify^9^, while the effect of evapotranspiration on precipitation is uncertain due to complex atmospheric processes and has been strongly debated^10^.

Previous studies showed discrepancies on feedback signs and intensities of the impact of soil moisture on precipitation. The study in Illinois is an interesting example. Findell and Eltahir^11^ stated that soil moisture in early summer was positively correlated with subsequent precipitation based on direct observations. However, Salvucci *et al*.^12^ argued that antecedent soil moisture had no effect on precipitation in Illinois. Furthermore, D’Odorico and Porporato^13^ found that soil moisture can affect the frequency of subsequent precipitation rather than the precipitation amount. Given these conflicting findings, further research is necessary to explore the soil moisture-precipitation coupling mechanism.

Due to the paucity of continuous observation data on soil moisture, previous studies are mainly based on climate models, such as the Atmospheric General Circulation Models (AGCMs)^14^, the Consortium for Small-Scale Modeling Model in Climate Mode (CCLM)^15^, the Weather Research and Forecasting (WRF) Model^16^, and the regional climate model (CLM)^17^. Such analyses could be limited by parameter settings and assumed behavior in models, which influences the simulated responsiveness^18,19^. To avoid this dependency, it is essential to conduct researches based on the observed data. Nowadays, remote sensing technology provides global available data of soil moisture, which facilitates this study.

However, it is difficult to establish causality based on observation data. In the literature, correlation analysis is commonly used to study causality^20,21^, but it is neither necessary nor sufficient for a causal link^22^. Granger causality, which can detect causality between variables based on predictability instead of correlation, is widely applied in econometrics^23^. However, it is applicable only to separable and purely stochastic systems, rather than dynamic systems^24^. Recently, a new approach known as convergent cross mapping (CCM) has been proposed to detect causality in dynamical systems based on empirical dynamics and Takens’ theorem^24^. CCM can also identify time-delayed causality and distinguish real bidirectional causality from the phenomenon of synchrony caused by strong unidirectional causality^25^. This method has been successfully applied in climate system, such as the interaction between temperature and greenhouse gases^26^, the relationship between galactic cosmic rays and temperature variations^27^, and the sensitivity of the carbon cycle to tropical temperature variations^28^. However, CCM method has not yet been applied in researches on soil moisture-precipitation interaction.

Based on remote sensing and re-analysis data (i.e., ESA CCI soil moisture, MODIS evapotranspiration, and CRU precipitation), we detect the causal effect of soil moisture on precipitation over low- and mid- latitude regions in the Northern Hemisphere using CCM method, and try to address three questions: (1) whether soil moisture can affect precipitation, (2) if so, whether there is a time delay in the causality, and (3) how this causality varies spatially over different climate conditions.

## Results

### The spatial patterns of soil moisture, evapotranspiration, and precipitation

**Figure 1:**
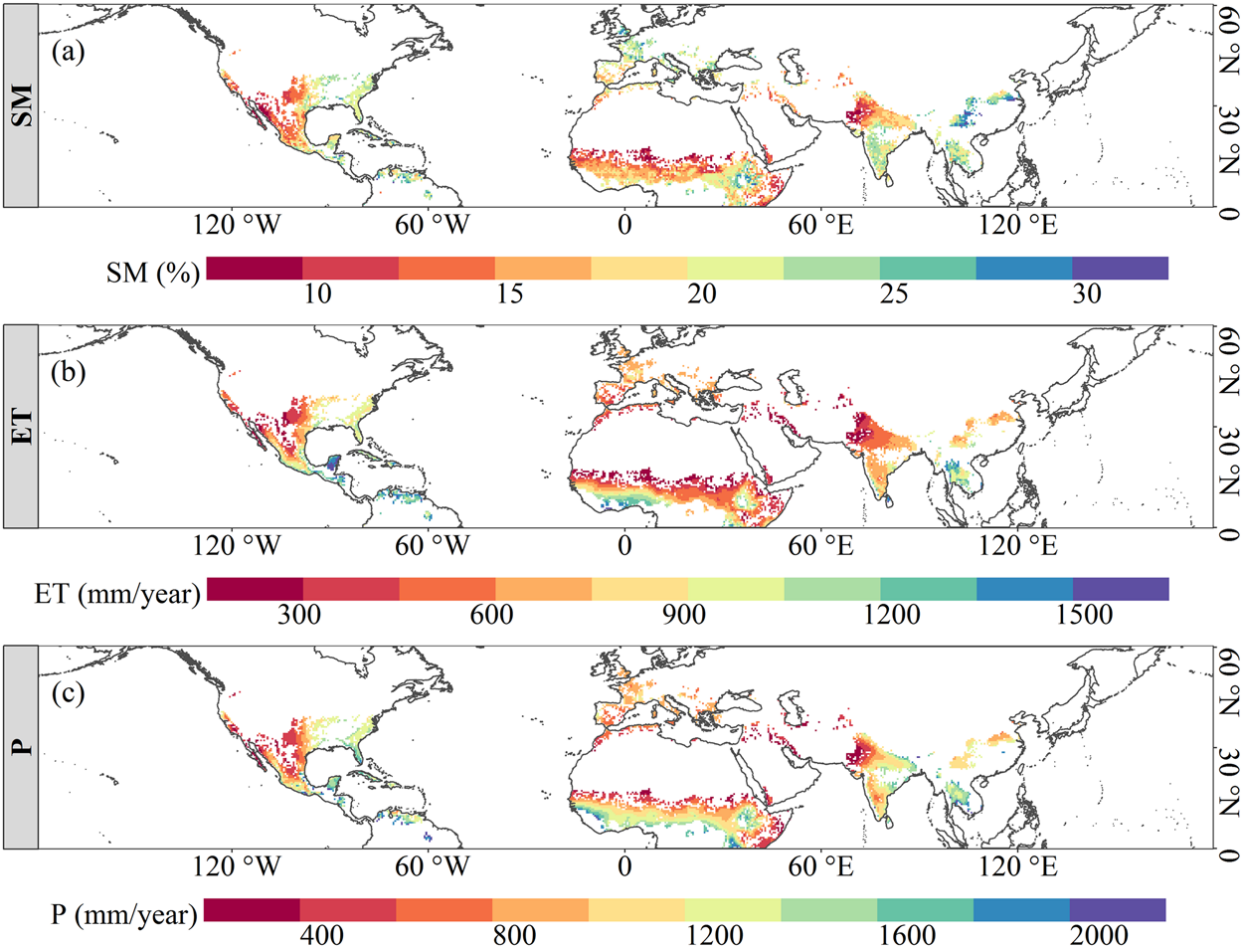
Spatial patterns of mean annual soil moisture, evapotranspiration and precipitation for the period 2010–2014 (Generated by free software R, https://www.R-project.org/).

This study focuses on low- and mid- latitude regions in the Northern Hemisphere (latitude 0°N~60°N). However, due to gaps in the soil moisture data, we extract grids with valid soil moisture data from January 2010 to December 2014 for analysis. Additionally, the study area is limited to regions with aridity index (AI) between 0.03 and 1 by excluding hyper-arid regions (AI < 0.03) with negligible soil moisture and hyper-humid regions (AI > 1) with negligible soil moisture changes. The finally study area is demarcated in color in Fig. 1.

Over the study area, soil moisture (SM) ranged from 7% to 37%, and exhibited an obvious spatial heterogeneity (Fig. 1). High soil moisture appeared in the Southeastern United States, France, Italy, Southern China, and Thailand, which indicates a relatively wet climate in these areas. In contrast, low soil moisture, signifying a dry condition, mainly distributed in the south-central United States, north-central Mexico, Pakistan, parts of India, and some equatorial African nations. Evapotranspiration (ET) and precipitation (P) showed a similar spatial pattern as soil moisture. Over regions with high soil moisture, evapotranspiration and precipitation were relatively high, and vice versa. There are also strong correlations among these three variables, with R = 0.897 between SM and ET, R = 0.894 between ET and P, and R = 0.795 between SM and P.

### Detecting causality in the soil moisture-precipitation process

**Figure 2:**
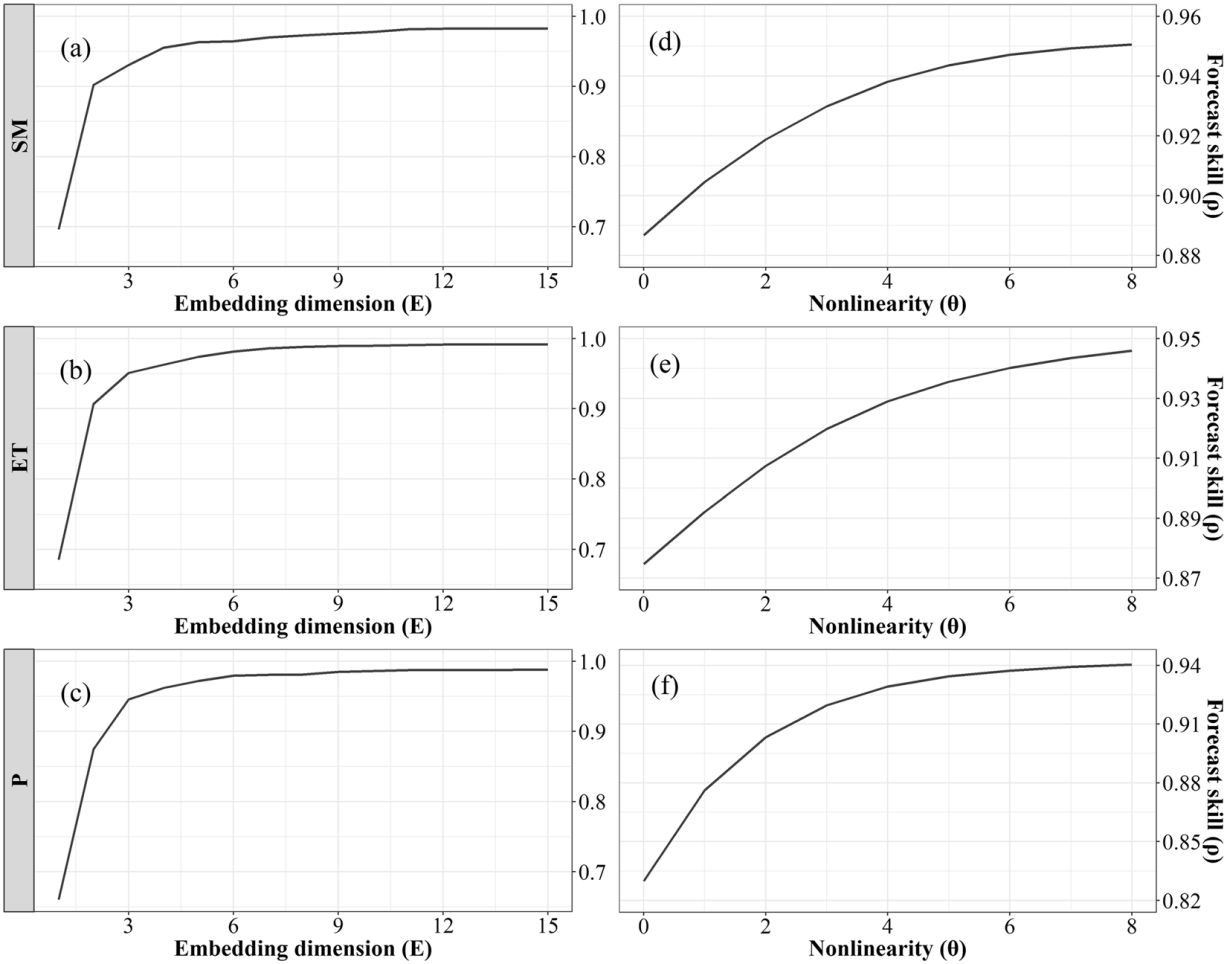
Forecast skill as a function of the embedding dimension (left column) and the nonlinearity index (right column) for soil moisture **(a**,**d)**, evapotranspiration **(b**,**e)** and precipitation **(c**,**f)** (Generated by free software R, https://www.R-project.org/).

In CCM method, the first step is to determine the optimal embedding dimension (E) which describes the size of the time windows used for prediction. Here, the simplex projection was applied to determine the optimal E for soil moisture, evapotranspiration, and precipitation, by using the forecast skill (ρ) as an indicator. Figure 2(a–c) presents the variation in the forecast skill as a function of E, which shows that the forecast skill approached a saturating status with the increasing of E. E near the saturation point is usually chosen as the optimal E. From this figure, a relatively high forecast skill was achieved with E = 6 for these three variables.

The second step of CCM is to use the S-map method to test the nonlinearity of the system. In this method, the nonlinear index (θ) is used to govern the weighting procedure, and the nonlinear dynamics system can be identified if the forecast skill improves as θ increases. In Fig. 2(d–f), the nonlinear models (θ > 0) gave better predictions than the linear model (θ = 0), which indicates statistical nonlinear behaviors in these three time series. Therefore, the CCM method can be applied to detect the causality between them.

**Figure 3:**
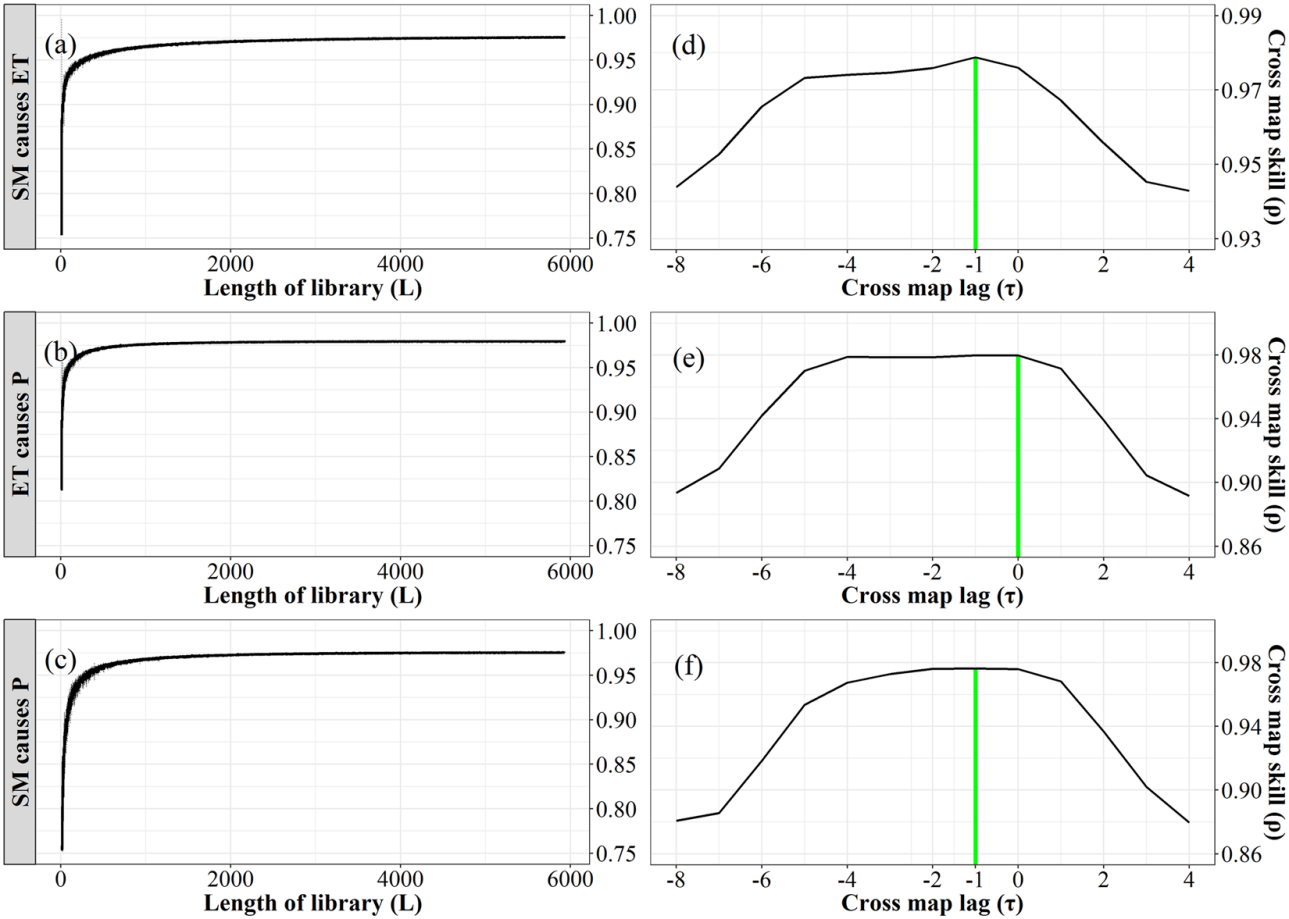
Cross map skill as a function of the length of library (left) and the cross map lag (right) for SM causing ET **(a**,**d)**, ET causing P **(b**,**e)** and SM causing P **(c**,**f)** (Generated by free software R, https://www.R-project.org/).

The effect of soil moisture on precipitation (SM-P) can be separated into two segments: the effect of soil moisture on evapotranspiration (SM-ET), and the effect of evapotranspiration on precipitation (ET-P). Here, CCM method was applied to analyze the causal relationships of SM-ET, ET-P, and SM-P, respectively. When applying CCM method, the cross map skill (ρ) is defined as the correlation coefficient between predictions and observations, and the length of library (L) refers to the number of historical observations. The causality is confirmed when ρ substantially increases with the increasing L. As presented in Fig. 3(a–c), for these three relationships, ρ increased with L and converged around L 1000, suggesting significant causal effects of SM on ET, ET on P, and SM on P, respectively. Additionally, CCM also showed a strong coupling within the system, as indicated by high cross map skills (ρ > 0.95).

In case of strong coupling, the phenomenon of synchrony must be addressed to infer the true causation. In this study, CCM method was repeated for different time lags (τ) to determine the optimal time lag which corresponds to the highest cross map skill. For real causality, the optimal cross map lag is non-positive. Conversely, the optimal cross map lag is positive. Figure 3(d–f) shows that the optimal effect of soil moisture on evapotranspiration occurred with a negative lag (τ = −1), meaning that an increase in soil moisture could not instantaneously translate into evapotranspiration increase. Evapotranspiration had a nearly instantaneous effect on precipitation, as the optimal cross map skill occurred with no time lag (τ = 0). CCM identified a true causal effect of soil moisture on precipitation with a negative optimal lag (τ = −1), suggesting that precipitation responded slowly to soil moisture change, due to a one month lag in converting soil moisture into evapotranspiration.

Figure 3(f) also shows that soil moisture had the strongest effect on precipitation with a one month lag, and the impact clearly decreased after four months, which reveals that soil moisture has the potential to predict precipitation at a sub-seasonal scale.

### Identifying regions with strong effects of soil moisture on precipitation

Identifying regions with strong effects of soil moisture on precipitation is important for designing the precipitation prediction system. Here the range of AI (0.03–0.1) was divided into 65 sub-ranges at intervals of 0.015, and the corresponding sub-regions were extracted from the study area. Then CCM method was applied to the data from each sub-region at different time lags to calculate the optimal effect of soil moisture on precipitation.

**Figure 4:**
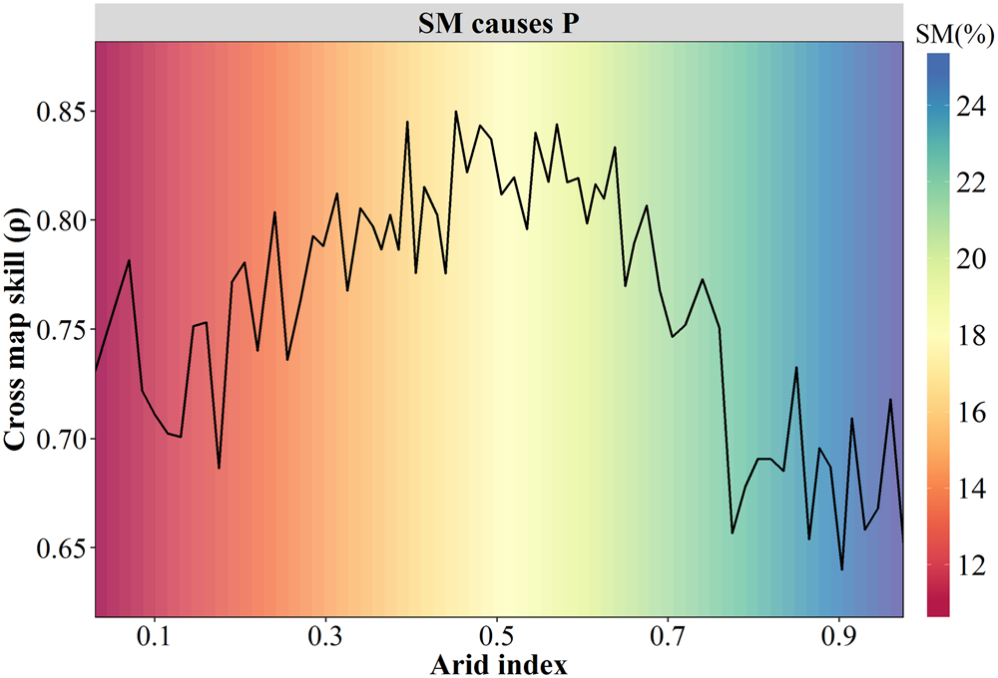
Variations of cross map skill across different climate regimes (Generated by free software R, https://www.R-project.org/).

Figure 4 displays the variation of cross map skill from dry to wet climate conditions. As AI increased, the cross map skill increased first and decreased afterward, with peaks in semi-arid and semi-humid area (0.3 < AI < 0.6), corresponding to the areas with soil volumetric moisture ranging between 15% and 20%.

**Figure 5:**
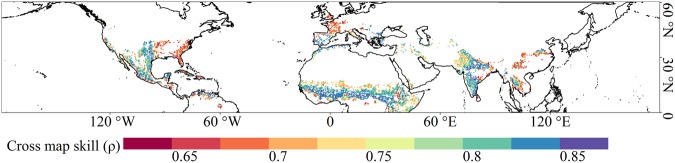
Spatial pattern of cross map skill (Generated by free software R, https://www.R-project.org/).

Figure 5 illustrates the spatial pattern of cross map skill over the study area. High cross map skills indicated strong effects of soil moisture on precipitation in the south-central United States, north-central Mexico, India, and some country of equatorial Africa.

## Discussions

Persistence is a distinctive attribute of soil moisture. A number of studies have investigated the soil moisture persistence, and indicated that the soil moisture persistence can span weeks to a couple of months^29–31^. Such persistence may turn out to be the main source of long-term weather prediction over mid-latitude continents^32^. However, the potential use of soil moisture persistence for precipitation forecasting has not been investigated as thoroughly. In this study, the persistence time in the effect of soil moisture on precipitation was quantified using CCM method, and the result shows that soil moisture has the potential to predict precipitation at a sub-seasonal scale. Furthermore, previous studies have demonstrated that surface soil moisture persistence was highly region and season dependent^33^. Hence, the persistence time in the effect of soil moisture on precipitation may vary with regions and seasons.

**Figure 6:**
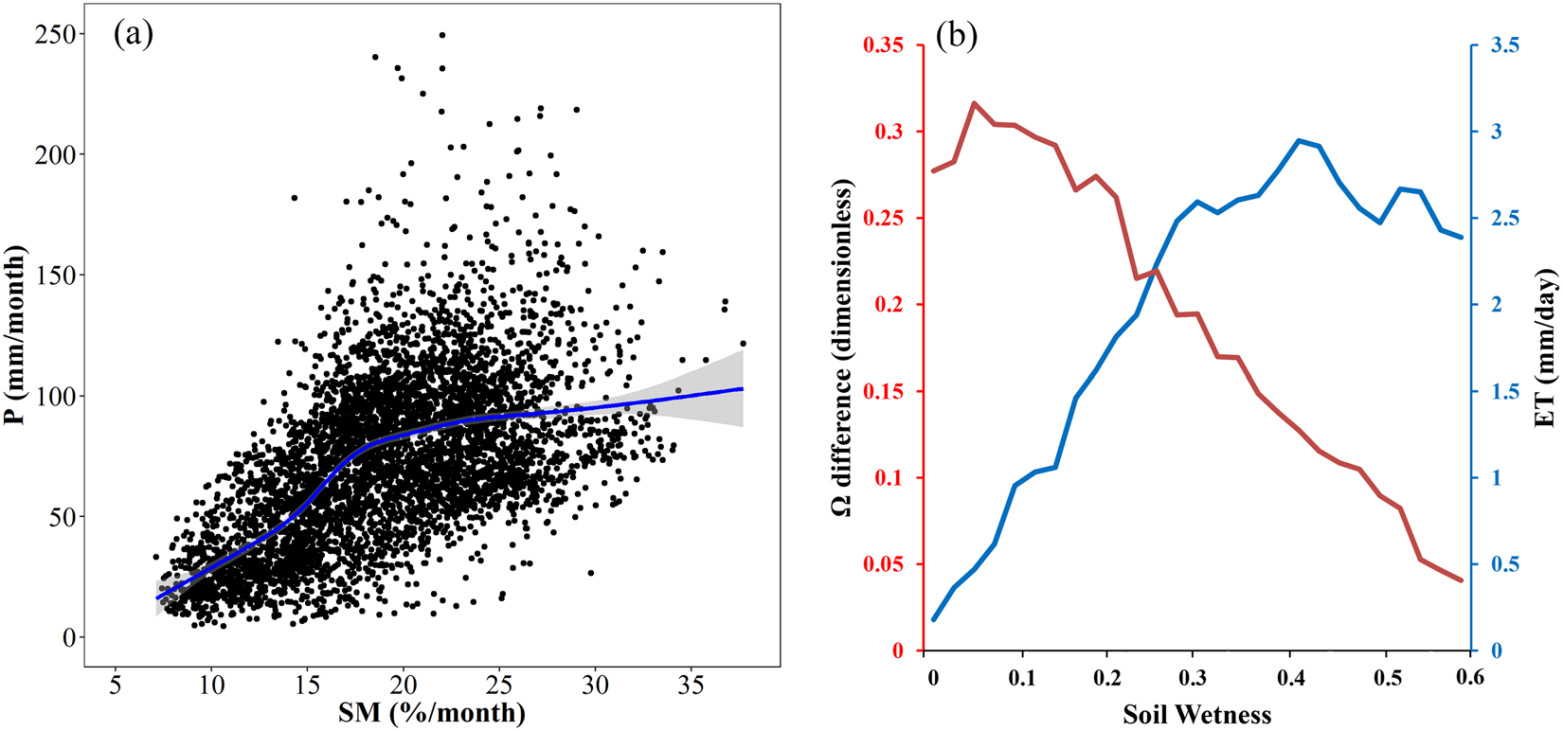
Relationships of soil moisture with precipitation **(a)** and evapotranspiration **(b)**. **(a)** presents scatterplots of mean monthly precipitation against soil moisture for all points over the study area, and the blue line is the fitted trend based on auto smoothing method, with shaded area denoting 95% confidence interval. **(b)** presents the variations of evapotranspiration and Ω difference (the fraction of variance in ET explained by soil moisture variations) with the increase of soil wetness (adjusted From Koster, R. D. *et al*. Regions of strong coupling between soil moisture and precipitation. Science 305, 1138–1140 (2004). Reprinted with permission from AAAS). (Generated by free software R, https://www.R-project.org/).

In addition, stronger effects of soil moisture on precipitation were detected in semi-arid and semi-humid areas compared to other regions (Fig. 4). To illustrate this phenomenon, Fig. 6(a) displays the variation of precipitation with soil moisture. The increasing rate of precipitation differed under different soil moisture conditions and exhibited an inverse tangent shape. The highest increasing rate was found in regions with soil moisture between 15% and 20%, which corresponds to regions with strong effects of soil moisture on precipitation (Fig. 4). Further, the relationship between soil moisture and evapotranspiration is depicted in Fig. 6(b). With soil moisture growth, the evapotranspiration (blue line) increased, while the dependency of evapotranspiration on soil moisture (Ω difference; red line) decreased. For hyper dry soil, both evaporation and its variation are too small to affect precipitation. As soil gets very wet and even approaches saturated, evapotranspiration is controlled by atmospheric condition rather than soil moisture, and thus soil moisture has little effect on precipitation^34^. Hence, the strong effect of soil moisture on precipitation mostly occurs in the transition between dry and wet soil, where a high sensitivity of evapotranspiration to soil moisture and a high variability of evapotranspiration are coexistent^35^. In addition, regions with stronger effects of soil moisture on precipitation identified by CCM method (Fig. 5) were in line with the results from Koster *et al*.^36^, that is, strong coupling regions were mainly distributed in the central Great Plains of North America, the Sahel, equatorial Africa, and India. This further increases the credibility of our results.

Finally, the soil moisture-precipitation relationship might be region dependent. In this study, a positive relationship between soil moisture and precipitation was discovered in the transitional regions with AI between 0.03 and 1, while Yang *et al*.^37^ observed a negative feedback over extreme dry and wet regions. This difference between the transitional regions and extremely dry/wet regions may arise from different relationships between soil moisture and evapotranspiration^38^. Vegetation can also affect precipitation through its influence on soil moisture and evapotranspiration^39^, thus there may be some discrepancies in soil moisture-precipitation relationship over different land types. Furthermore, the soil moisture-precipitation feedback at different spatiotemporal scales can also make a difference. For instance, Hohenegger *et al*.^40^ found a positive feedback at a 25-km spatial resolution and a negative feedback at a 2.2-km spatial resolution based on hourly data, and Guillod *et al*.^41^ suggested positive temporal effects and negative spatial effects of soil moisture on afternoon rainfall at a spatial resolution of 1.25 degree. Hence, it should be a future hotspot to explore the soil moisture-precipitation coupling mechanism in different regions and spatiotemporal resolutions.

## Conclusions

In this study, CCM method was used to detect causality between soil moisture and precipitation over low- and mid- latitude regions in the Northern Hemisphere. A strong causality between SM-ET, ET-P, and SM-P was detected with cross map skill more than 0.95, which supports the assertion that soil moisture affects precipitation through evapotranspiration. The optimal cross map skill for soil moisture affecting precipitation occurred with a lag of one month, and then substantially decreased after four months, suggesting that soil moisture is potential to predict precipitation at a sub-seasonal scale. As the climate changed from dry to wet, the effect of soil moisture on precipitation increased first and then decreased, with the strongest effects appearing in semi-arid and semi-humid area, such as the south-central part of the United States, north-central of Mexico, India, and some country of equatorial Africa.

Although this study successfully detected the effect of soil moisture on precipitation at both temporal and spatial scales, there are still some spaces for improvement. For instance, we only detected the causal effect over regions with valid soil moisture data, while there are large areas without valid soil moisture data over low- and mid- latitude regions in the Northern Hemisphere. In addition, this study only considered the local effect rather than the remote effect, and the mechanisms for the remote effect need to be explored in the future.

## Methods

### Precipitation data (P)

Monthly precipitation data are collected from the Climate Research Unit time series data version 4.0 (CRU Ts4.0), for the period from January 2010 to December 2014. This dataset is gridded onto a 0.5° network, based on analysis of more than 4,000 weather station records^42^.

### Soil moisture data (SM)

This study uses the soil moisture dataset released by the European Space Agency (ESA) Climate Change Initiative (CCI) program^43^. Version CCI v03.3 contains an active data set, a passive data set and a combined data set. The combined data set used in this study is derived from three active (AMI-WS, MetOp-A ASCAT, MetOp-B ASCAT) and seven passive (SMMR, SSM/I, TMI, WindSat, AMSR-E, AMSR2, SMOS) microwave sensors. The dataset has a temporal resolution of one day and a spatial resolution of 0.25°, and provides surface soil moisture information in volumetric units (m^3^·m^−3^). Monthly soil moisture data are calculated from ESA CCI daily data. To guarantee the accuracy of monthly average values, the averaging conversion is only applied to pixels where valid data have a length of more than 15 days a month^44^. The monthly soil moisture data are then averaged spatially to a resolution of 0.5°.

### Evapotranspiration data (ET)

Evapotranspiration data downloaded from MODIS (MOD16) for the period January 2010 to December 2014 and at a spatial resolution of 0.5° are used in this study (http://files.ntsg.umt.edu/data/NTSG_Products/MOD16/MOD16A2_MONTHLY.MERRA_GMAO_1kmALB/GEOTIFF_0.5degree/). The MOD16 ET is calculated based on the Penman-Monteith theory, which performs well in generating global evapotranspiration data^45^.

### Aridity index (AI)

To understand the dependency of precipitation on soil moisture across different climate regimes, the mean global aridity index (AI) from CGIAR-CSI (http://www.cgiar-csi.org) is used for climate classification. The AI is calculated as:

1 $${\rm{AI}}={\rm{MAP}}/{\rm{MAE}}$$

where MAP is the mean annual precipitation, and MAE is the mean annual potential evapotranspiration.

According to the United Nations Environment Programme (UNEP 1997), there are five climate classes: hyper-arid (AI < 0.03), arid (0.03 < AI < 0.2), semi-arid (0.2 < AI < 0.5), semi-humid (0.5 < AI < 0.65), and humid (AI > 0.65).

### Convergent cross mapping (CCM)

It is a new approach for detecting the causal relationships in nonlinear dynamical systems. CCM is based on the Takens’ theorem, which states that in a multi-dimensional dynamical system, the essential information can be retained in the time series of any single variable of the system^24,46^. In CCM, causality is detected by measuring the extent to which the time series historical record of one variable can reliably estimate states of the other variable. That is, if variable X is influencing Y, then, based on the generalized Takens’ theorem, the causal variable X can be recovered from the historical record of the affected variable Y. The cross map skill is defined as the correlation coefficient ρ between predictions and observations of X. If the cross map skill increases with the length of the time series and convergence is present, then the causal effect of X on Y can be inferred. More details can be found in three one-minute animations from the supplementary material of Sugihara *et al*.^24^ (http://science.sciencemag.org/content/suppl/2012/09/19/science.1227079.DC1). CCM can also distinguish the true bidirectional causality from the phenomenon of synchrony resulting from extremely strong unidirectional forcing^24^. In case of synchrony, although variable X does influence Y, and Y has no effect on X, CCM can detect causal relationships in both directions. To resolve this problem, Hao *et al*.^25^ proposed an extension to CCM by considering time lags, i.e., a non-positive lag for optimal cross mapping for the true causal direction while a positive lag for the false causal direction.

In this study, the CCM analysis was implemented by using the free rEDM package^47^ and multispatialCCM package^48^ within the R language environment^49^, and the analysis was based on monthly data of grids in the study area for the period from January 2010 to December 2014. The implementation procedure includes following three steps:

1. Identify the optimal embedding dimension (E) using simplex projection. In this algorithm, the ability of one variable to predict its own dynamics is estimated using different embedding dimensions^50^. The correlation coefficient ρ between predictions and observations represents the forecast skill, and the optimal embedding dimension corresponds to the highest forecast skill.
2. Test the nonlinearity using the S-map test (sequential locally weighted global linear maps). The S-map method can describe the dynamics by fitting local linear maps^51^, and uses a nonlinearity index θ to govern the weight of points when fitting the local linear map. When θ = 0, all points are weighted equally, and the model corresponds to a simple linear model, and when θ > 0, the nearby points are weighted greater, and the forecast becomes more nonlinear. If the best forecast skill appears in a linear model (θ = 0), the system has only linear dynamics. In contrast, if forecast skill ρ improves with increasing θ, the system is nonlinear.
3. Calculate the cross map skill for different time lags. In the CCM method, the cross map skill (ρ) varies as a function of the length of the library (L), which represents the number of observations in the composite time series collapsing from multiple plots. The CCM method is executed at different time lags (τ) to distinguish the true bidirectional causality from the synchrony.

## Data Availability

No datasets were generated during the current study.
